# Assessment of Visual Fatigue Caused by Eye-Controlled Interaction Based on Task Performance and Pupillary Response with GBDT-LR

**DOI:** 10.3390/s26175507

**Published:** 2026-08-30

**Authors:** Hongwei Niu, Ziyi Zhao, Mingyu Ai, Xiaonan Yang, Xuan Zhang, Haonan Fang

**Affiliations:** 1 Industrial and Systems Engineering Laboratory, School of Mechanical Engineering, Beijing Institute of Technology, Beijing 100081, China; nhwfend@bit.edu.cn (H.N.); 1120223309@bit.edu.cn (Z.Z.); 3220230332@bit.edu.cn (M.A.); yangxn@bit.edu.cn (X.Y.); 3120235091@bit.edu.cn (H.F.); 2 Yangtze Delta Region Academy, Beijing Institute of Technology, Jiaxing 314019, China; 3 Key Laboratory of Industry Knowledge & Data Fusion Technology and Application, Beijing Institute of Technology, Beijing 100081, China; 4 School of Design & Arts, Beijing Institute of Technology, Beijing 100081, China

**Keywords:** eye-controlled interaction, visual fatigue, eye tracking, Bayesian optimization, GBDT-LR

## Abstract

**Highlights:**

This study proposes a fusion of subjective and objective measures for visual fatigue induced in eye-controlled interaction by integrating eye movement features and task performance indicators.

**What are the main findings?**

- A GBDT-LR model optimized via Bayesian optimization achieved an assessment accuracy of 89.79%. Its performance was comparable to that of the best-performing GBDT and substantially higher than those of LR, SVM, RF, and RF-SVM.
- Six non-redundant indicators spanning blink, fixation, pupil, and task performance dimensions were selected to characterize visual fatigue.

**What are the implications of the main findings?**

- The lightweight pipeline provides a practical foundation for real-time fatigue monitoring and user experience optimization in eye-controlled systems.
- The method offers a non-invasive, easily deployable tool for assessing visual fatigue in eye-controlled interaction systems.
- The framework supports future adaptive interfaces that optimize user experience and promote sustainable use of eye-control technology.

**Abstract:**

Assessing visual fatigue is crucial in eye-controlled interaction. Traditional methods are either overly subjective or rely on highly invasive, costly equipment and complex procedures that require expert supervision. This study proposes a machine-learning-based approach for visual fatigue assessment. Data collection employs non-intrusive, easily monitored eye-tracking to capture ocular eye movement data and task performance data, while subjective questionnaires label fatigue states. For feature selection, participant-level Wilcoxon signed-rank tests with Benjamini–Hochberg FDR correction were used to identify fatigue-related indicators, and a redundancy-removal step based on Spearman correlation yielded a final set of six non-redundant features. For the assessment method, we introduced a gradient boosting decision tree–logistic regression (GBDT-LR) model whose hyperparameters are optimized via Bayesian optimization. All models were evaluated under a unified 5-fold stratified cross-validation framework with within-fold standardization and nested hyperparameter tuning. Results indicate that this model can effectively predict the state of visual fatigue. Compared with the performance of five other models—gradient boosting decision tree (GBDT), logistic regression (LR), support vector machine (SVM), random forest (RF), and RF-SVM—the proposed GBDT-LR model achieved an assessment accuracy of 89.79%, demonstrating strong predictive performance. This study provides an effective method for predicting visual fatigue in eye-controlled interaction, laying a research foundation for optimizing the user experience of eye-controlled interaction and promoting the sustainable development of eye-control technology.

## 1. Introduction

Eye-controlled interaction is an innovative human-computer interaction method that utilizes eye movements to interact with computers. This method boasts several advantages, including its natural and intuitive nature, rapid and precise response capabilities, and the absence of physical contact [1,2]. It is widely applied across various domains, such as computer operations, virtual reality, gaming, medical rehabilitation, and more, offering enhanced convenience and novel experiences to users [3,4,5,6].

However, eye-controlled interaction scenarios are often task-driven, imposing a significant load on the eyes due to numerous specific, constrained actions combined with prolonged usage and visual stimuli. This excessive physiological load induces visual fatigue, a condition marked by decreased visual system performance and overall discomfort [7]. Visual fatigue can adversely affect user experience and operational performance during eye-controlled interactions [2].

To measure and assess the state of visual fatigue, it is necessary to propose an effective measurement based on the state of the operator. Following ISO 9241-303 [8], visual fatigue is conceptualized as a functional impairment of the visual system resulting from prolonged visual task performance, distinct from transient eye strain (a subjective sensation) and visual discomfort (a general bad experience). Assessments can be broadly categorized into subjective evaluations, objective measurements, and task assessments. Initially, visual fatigue was assessed primarily through subjective questionnaires, such as the Visual Fatigue Scale (VFS) [9], Simulator Sickness Questionnaire (SSQ) [10], and others developed by researchers like James [11] and Shibata et al. [12]. Although these subjective scales are easy to administer, they are influenced by personal perceptions and lack objectivity and real-time feedback capabilities. Therefore, objective measurement is carried out by measuring changes in physiological parameters, such as electrocardiography (ECG) [13], electro-oculography (EOG), electromyography (EMG), and electroencephalography (EEG) [14]. However, the acquisition of objective indicators is either overly invasive and difficult to collect, or the process is too cumbersome and must be carried out by trained personnel. Task performance changes can also serve as indicators of visual fatigue. For example, Naeeri evaluated pilot fatigue using psychomotor vigilance tasks (PVT) [15]. Since visual fatigue tends to most easily affect eye-related indicators among objective metrics, and considering the comprehensiveness and generalizability of measurements, this study simultaneously adopts three methods to ensure accurate and comprehensive visual fatigue assessment: a subjective questionnaire, an objective eye movement measurement method, and a task performance measurement.

As machine learning methods become increasingly mature, they have been widely used to develop predictive models for fatigue. These models utilize features extracted from subjective reports or objective measurements and learn to correlate them with fatigue degrees. In this study, we propose a GBDT-LR model optimized with a Bayesian algorithm to predict visual fatigue in eye-controlled interaction. By integrating eye movement characteristics and task performance data, we aim to develop a comprehensive assessment model. For data collection, we adopt a comprehensive approach that integrates both subjective and objective metrics: subjective questionnaires are used to label fatigue states, while an eye tracker is employed to capture ocular eye movement data and task performance data indices that reflect visual fatigue. This data collection method is non-invasive and simple to operate, making it more accessible for widespread use. For the assessment method, the GBDT-LR model combines the strengths of gradient boosting decision trees (GBDTs) and logistic regression (LR), offering robust predictive capabilities by capturing complex data patterns and providing interpretable results [16]. Furthermore, the model’s hyperparameters are optimized using the Bayesian optimization algorithm, which enhances model performance and accuracy. Studies have demonstrated the effectiveness of Bayesian optimization in improving the predictive accuracy and computational efficiency of machine learning models [17]. Our model’s performance is compared with five other models: GBDT, LR, support vector machine (SVM), random forest (RF), and RF-SVM, demonstrating its superiority in accurately predicting visual fatigue. This study contributes to improving user experience and promoting sustainable development in eye-controlled interaction technologies.

The rest of this paper is organized as follows. Section 2 reviews related work. Section 3 describes the experimental procedure and data acquisition. The study’s results and discussion are presented in Section 4 and Section 5, respectively. Section 6 summarizes the conclusions of this paper.

## 2. Related Work

### 2.1. Eye-Controlled Interaction

Eye-controlled interaction technology represents a significant advancement in the field of human-computer interaction. It offers a hands-free and intuitive method of engaging with digital interfaces [2]. In complex task scenarios, eye-controlled interaction can fully leverage its characteristics, liberate the operator’s hands, and complete tasks without contact, greatly improving operational efficiency and accuracy [18,19,20]. This technology is particularly beneficial for individuals with severe motor disabilities, as it allows them to interact with computers using only their eye movements, which they can often control despite limitations in other motor functions [21]. People with conditions such as quadriplegia, amyotrophic lateral sclerosis, and cerebral palsy can leverage eye-controlled systems to perform tasks that would otherwise be unattainable. Huang et al. proposed a novel free-eye drawing system called EyeCompass, which employs eye-controlled interaction for drawing with the gaze, offering significant value for individuals with motor disabilities [22]. Rajanna et al. presented a dwell-free, multimodal, gaze typing system that uses a supplemental foot input to select characters [23]. With this design, a user focuses on the target character with their gaze and selects it with the foot input. The study provides interactive means of text input for individuals with injuries and disabilities due to the environment and with sports injuries (physical injuries and disabilities) due to birth or injury. Eye-controlled interaction often comes with visual fatigue, which not only reduces comfort and productivity but also poses a risk to people’s health [24,25,26]. Considering the impact of visual fatigue, Wambecke et al. presented an interaction technique to select on-screen objects and navigate menus through the synergistic use of eye-gaze and thumb-to-finger micro-gestures; the results show that the new interaction technique tends to yield better time performance when participants additionally need to treat high-priority tasks in parallel and induces less fatigue [27]. Therefore, it is very important to explore the mechanisms by which eye-controlled interaction affects visual fatigue and to predict the onset of associated visual fatigue. This will support the widespread use of eye-controlled interaction.

### 2.2. Visual Fatigue Assessments

A broad range of subjective and objective indicators have been employed to quantify visual fatigue. On the subjective side, Jiang et al. drew upon human subjective research to develop learning-to-rank techniques for visual comfort assessment, applying these subjective metrics to the evaluation of visual fatigue in stereoscopic images [28]. On the objective side, Shi et al. measured visual discomfort using eye movement and heart rate signals [29]; Song et al. extracted and classified features from EEG signals to assess visual fatigue [30]; Chen et al. relied on a set of 13 eye-related features to measure visual fatigue in virtual reality environments [31]; Wang et al. proposed an approach based on five ophthalmic parameters and three physiological signals to evaluate visual fatigue under LED illumination [32]. However, methods that rely solely on subjective or objective indicators cannot fully assess the state of visual fatigue.

The physiological mechanisms underlying visual fatigue provide a necessary theoretical basis for selecting and interpreting eye movement indicators. Prolonged near-work on a screen induces accommodative microfluctuations and vergence-accommodation conflict, which increase the load on the ciliary muscle and extraocular muscles and may manifest as prolonged fixation duration, reduced saccadic amplitude, and altered pupillary responses. The pupil, in particular, is regulated by the antagonistic interplay between the sympathetic (dilating) and parasympathetic (constricting) autonomic pathways; sustained cognitive and visual load can shift this balance, producing measurable changes in mean pupil diameter and pupil diameter variability. Meanwhile, reduced blink rate and increased PERCLOS (the proportion of time the eyelids cover more than 80% of the pupil) reflect tear-film instability and increased corneal exposure, both of which are associated with ocular surface fatigue. Consequently, eye movement features can be mapped to specific physiological processes: fixation and saccade metrics reflect oculomotor control and accommodative/vergence load; blink-related metrics reflect ocular surface status and compensatory mechanisms; and pupillary metrics reflect autonomic arousal and cognitive effort. Without such mechanistic grounding, feature selection risks appearing purely empirical.

Among studies that integrate subjective and objective metrics, Jia et al. combined subjective questionnaires with ophthalmic parameters, EEG, and ECG to model visual fatigue in 2-D, 3-D, and augmented 3-D reading environments [13]. Yet the cumbersome acquisition of EEG and ECG data limits the method’s generalizability. Wang et al. incorporated eye movement parameters (pupil size and blink frequency), subjective ratings, LCD luminance characteristics, and environmental factors to assess visual comfort [33], but their analysis focused on eye movement factors without examining the relative importance of the different indicators. Moreover, eye-controlled interaction requires the eye to act as an input modality, whereas prior research merely required passive viewing. Consequently, findings from earlier studies cannot be directly extrapolated to the visual fatigue induced by eye-interactive tasks. This underscores the urgent need to develop assessment methods tailored specifically to eye movement interaction scenarios.

Recent advances in adaptive eye-tracking interfaces further motivate this need. Systems that dynamically adjust dwell time, target size, or gaze-contingent feedback based on estimated fatigue can reduce visual load and prolong comfortable interaction. However, such adaptations require accurate, real-time fatigue estimation, which remains underdeveloped for eye-controlled interaction. In addition, individual differences in fatigue susceptibility—related to age-related accommodative amplitude decline, pupillary light-reflex changes, and baseline dry-eye status—mean that models trained on homogeneous young adults may not generalize to older or clinical populations. Therefore, this study adopts subjective questionnaires to measure subjective data on visual fatigue, utilizes eye-related data collected by the eye tracker, and combines task performance to comprehensively measure and evaluate visual fatigue during the eye-controlled interaction process.

### 2.3. Machine Learning Algorithms for Fatigue Assessment

In recent years, machine learning methods have been increasingly used to develop predictive models for fatigue. These models utilize features extracted from subjective reports, such as questionnaires, and objective measurements, such as physiological signals and task performance data, to learn to correlate these features with fatigue degrees. Common approaches include LR, GBDT, SVM, and RF. Ries et al. used a functional logistic regression model to predict physical fatigue by analyzing heart rate characteristics, emphasizing that the model provides good predictive and interpretable parameters for practical applications [34]. Similarly, Lee et al. used stepwise logistic regression to predict fatigue in construction workers, considering heart rate variability measurements, standard deviation of Normal to Normal (NN) intervals, and low-frequency range (LF) power for fatigue assessment [35]. Hu et al. used EEG signals as the main feature as input for GBDT and compared it with three state-of-the-art classifiers, k-nearest neighbor, support vector machine, and neural network, demonstrating the effectiveness of GBDT in predicting driver fatigue and significantly improving assessment performance [36]. Xiong et al. applied SVM to classify fatigue levels based on features extracted from the combined processing of entropy EEG data, demonstrating that SVM can provide high accuracy in fatigue detection by effectively separating the data into different classes [37]. Zhou et al. designed an improved RF model to analyze the percentage of eyelid closure (PERCLOS) over pupil time, blink frequency (BF), yawn frequency (YF), and head nod frequency (HNF) extracted from video images. This method effectively predicts fatigue by capturing important features from data, demonstrating the powerful capabilities of RF [38].

Beyond conventional classifiers, deep-learning architectures have been increasingly explored for eye movement and physiological signal analysis. Recurrent neural networks (RNNs) and long short-term memory (LSTM) networks are particularly well suited to modeling the temporal dynamics of eye-tracking sequences, because they can capture dependencies across consecutive fixations, saccades, and pupil diameter fluctuations [39]. More recently, convolutional neural networks (CNNs) and attention-based transformers have been applied to raw eye movement traces for fatigue, workload, and disease classification [40]. These approaches can learn hierarchical spatial-temporal representations without hand-crafted features; however, they typically require large, densely sampled datasets and extensive hyperparameter tuning, and their ‘black-box’ nature reduces interpretability. In the present study, we prioritize a lightweight and interpretable pipeline that can be validated on a modest sample (48 participants); nevertheless, sequence-aware and deep-learning extensions are planned as the dataset grows.

For the present study, we selected a GBDT-LR hybrid model for three reasons. First, the dataset is relatively small (48 participants), making data-efficient, strongly regularized tree ensembles preferable to deep networks that typically require larger temporal sequences. Second, eye-tracking features are heterogeneous in scale and contain non-linear interactions; GBDT automatically discovers discriminative feature combinations, and its one-hot-encoded leaf indices provide an effective non-linear transformation for the subsequent LR layer. Third, the resulting LR coefficients offer interpretability, which is important for understanding the relative contribution of oculomotor, pupillary, and task performance indicators. Although sequence-based models could be explored in future work with larger longitudinal recordings, the GBDT-LR approach provides a pragmatic balance of predictive accuracy, data efficiency, and interpretability for the current eye-controlled interaction scenario.

Although machine learning algorithms are being increasingly applied to predict physical fatigue in users, it remains to be seen whether they can also perform well in predicting visual fatigue. Based on the good performance of the GBDT-LR algorithm in assessment [41], this algorithm will be used to establish a visual fatigue assessment model and compared with other machine learning algorithms used to establish fatigue models (GBDT, LR, SVM, RF, RF-SVM). It is important to study the capability of the GBDT-LR algorithm in visual fatigue assessment and to establish a more accurate visual fatigue assessment model.

## 3. Experimental Design and Data Collection

### 3.1. Experiment Setting

#### 3.1.1. Experimental Objectives and Materials

**Figure 1:**
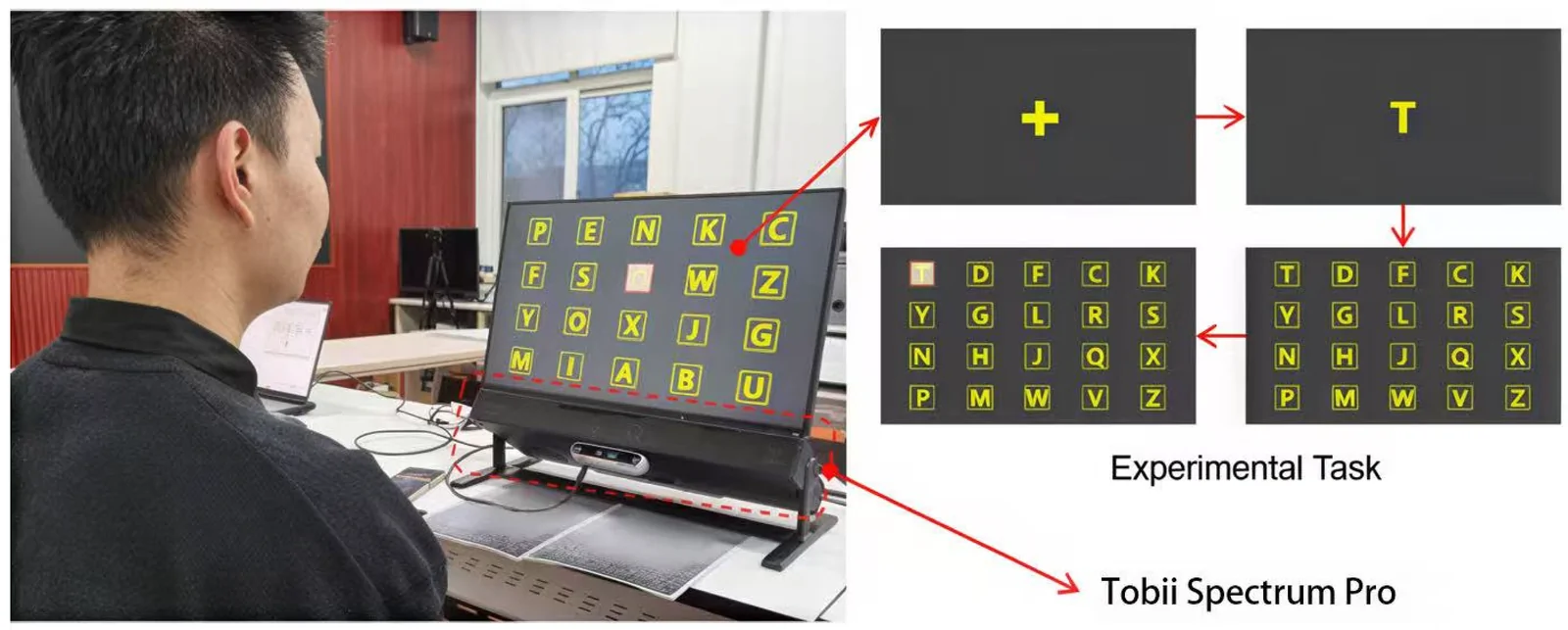
Experimental demonstration diagram.

This study conducted an experiment to induce visual fatigue through eye-controlled interaction, aiming to measure subjective data, objective eye-related data, and task performance during the eye-controlled interaction process and thereby assess the state of visual fatigue. The eye movement data were collected using the screen-based eye tracker Tobii Pro Spectrum (as shown in Figure 1), configured at a sampling rate of 150 Hz. The Tobii Pro Spectrum features a high sampling rate of up to 1200 Hz with 0.17° of spatial accuracy, capable of capturing 1200 eye images per second, and it has a wide compatibility range regardless of whether participants wear vision correction glasses.

**Figure 2:**
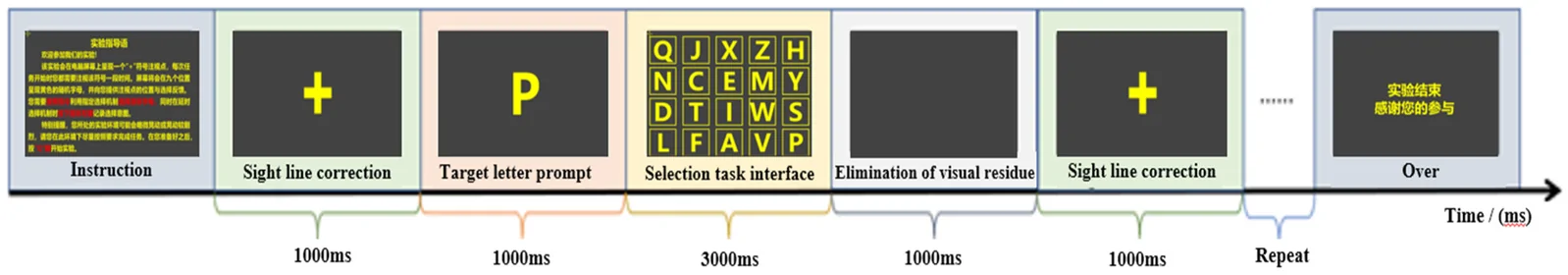
Task flow The Chinese text shown in the figure represents the experimental instructions and the reminder of the end of this experiment, which was presented to the participants.

The experimental task was a selection task, as illustrated in Figure 2. It began with an explanatory introduction, followed by a central “+” displayed for 1000 milliseconds to adjust the participants’ visual focus. Subsequently, the letter chosen by the participant was displayed, and they were required to accurately select the correct letter from a selection interface of 20 letters. Successful selection was acknowledged with animated feedback, indicating successful recognition. This process was iterated continuously until the participant felt dryness, eye pain, blurred vision, itching or stinging, headache, or eye pressure and voluntarily terminated the task. To ensure the authenticity of visual fatigue, participants were informed of these associated symptoms before the experiment. Based on this information, they self-assessed their level of visual fatigue.

To protect participants’ well-being, the maximum session duration was capped at 40 min, and experimenters monitored participants continuously; any participant who reported severe discomfort was allowed to stop immediately. Furthermore, we ensured that each participant engaged in each task for at least 5 min.

Environmental conditions were held constant across sessions: the laboratory was illuminated by overhead fluorescent lamps at approximately 300 lux (measured at the desk surface), window curtains were drawn to minimize daylight variation, the monitor refresh rate was 60 Hz, the screen brightness was fixed at approximately 120 cd/m^2^, and participants were seated at a viewing distance of approximately 60 cm from the screen. These settings conform to typical visual display terminal (VDT) work conditions and were chosen to approximate real-world eye-controlled interaction scenarios.

#### 3.1.2. Participants and Experimental Procedure

A total of 48 participants were invited to take part in the experiment (38 males and 10 females; with an average age of 22.29 years and a standard deviation of 1.50 years). These were college students and graduate students with no eye diseases, not wearing contact lenses, colored contact lenses, or fake eyelashes, and with sufficient sleep. The data collected from all 48 participants were valid.

The entire experiment consisted of two phases: preparation and implementation. During the preparation phase, participants were informed of the purpose and content of the experiment and then asked to sign an informed consent form and fill out a personal information questionnaire. In the implementation phase, participants first underwent a 9-point eye calibration, followed by a brief pre-experiment practice session to familiarize themselves with the experimental task. After the pre-experiment, participants were required to complete a subjective questionnaire before the formal experiment began, which was preceded by another eye calibration. After the formal experiment, participants submitted a new subjective questionnaire.

**Figure 3:**
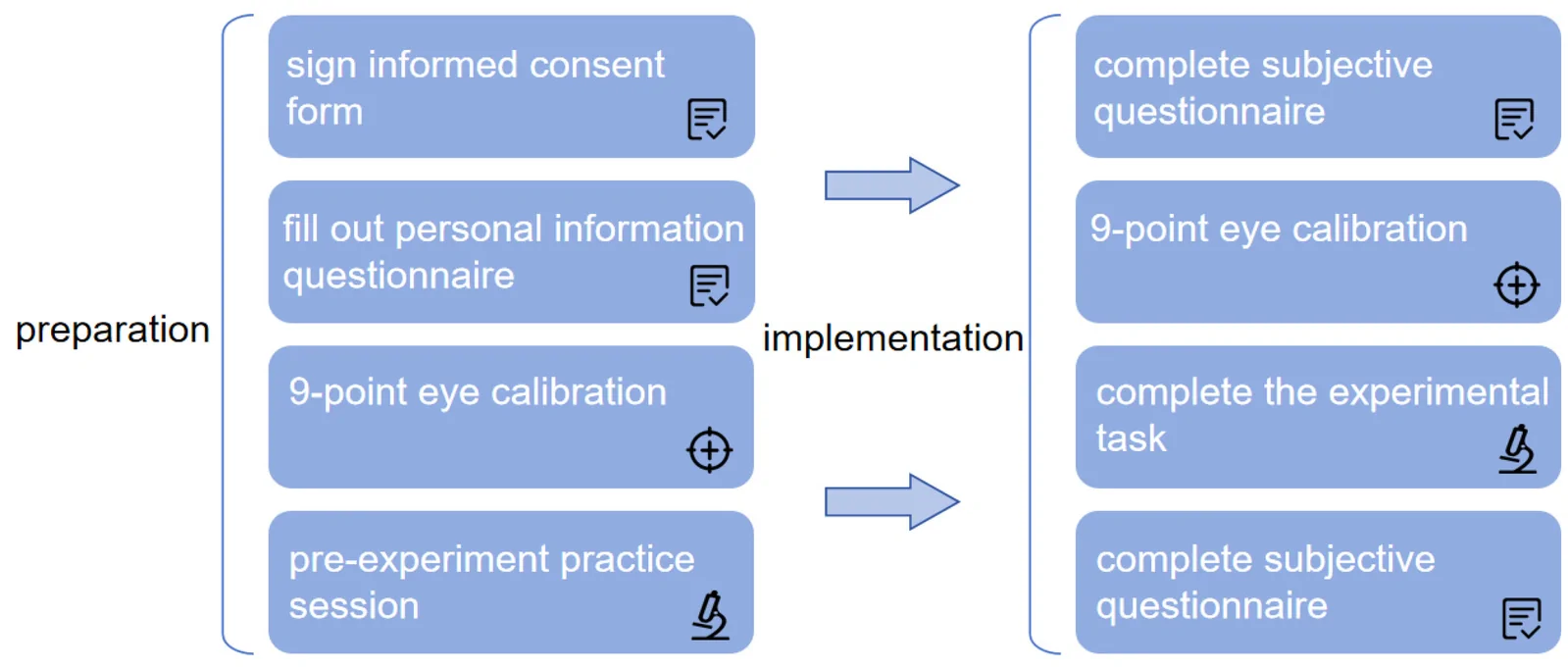
Experimental flowchart.

To mitigate potential learning effects, all participants completed the same standardized practice session before data collection, and the task rules remained unchanged throughout the formal experiment. The experimental flowchart is shown in Figure 3.

### 3.2. Measured Feature Set

The subjective questionnaire measured subjective visual fatigue, with questions 1 to 3 (How tired are your eyes; How clear is your vision; How do your eyes feel) used to assess subjective ocular symptoms, and questions 4 to 7 (How tired is your back; How tired is your neck; How severe is your headache; How sleepy do you feel) used to assess physical and psychological discomfort. Each question is scored from 0 to 4, with increasing levels of fatigue, and participants choose a score for each question. The final scores of the seven questions are added together to serve as the total score for subjective visual fatigue. The higher the subjective visual fatigue score, the greater the degree of visual fatigue.

The eye movement data was collected using the Tobii Pro Spectrum eye tracker. Real-time raw eye movement data, including timestamps, user coordinates of the left and right eyes, screen coordinates of the left and right eyes, three-dimensional coordinates of the eye tracker for both eyes, pupil diameter of the left and right eyes, and various data validity indicators, were obtained through Python 3.10.9 by invoking the Tobii Pro SDK. Then, based on the I-VT algorithm, eye movement characteristics were extracted, and 17 feature data were obtained, including number of fixations, total fixation duration, average fixation duration, first fixation duration, number of saccades, total saccade duration, average saccade duration, average saccade amplitude, average saccade velocity, number of blinks, blink frequency, total blink duration, average blink duration, percentage of eyelid closure over the pupil over time (PERCLOS), mean pupil diameter, standard deviation of pupil diameter, and coefficient of variation of pupil diameter.

For task performance data, it mainly included selection reaction time and cumulative selection accuracy. Selection reaction time refers to the time it takes from the presentation of a stimulus to the first entry of the target letter range in an experiment. Because the selection task actually involves the process of visual search, the area where the gaze first enters may not necessarily be the location of the target letter, so the selection reaction time here emphasizes the time when the target range is first searched. The definition of cumulative selection accuracy is the percentage of correct choices made up to the current number of trials. It should be noted that if the first selection is incorrect, it will have a significant impact on cumulative selection accuracy. So, to balance the cumulative selection accuracy and enable it to effectively evaluate the performance of choices, regardless of whether the first choice is correct or not, it is considered a correct choice. That is to say, the first trial is treated as 100% correct because the selection task involves visual search and the first gaze entry may not land on the target; forcing the initial accuracy to 100% prevents a single initial error from disproportionately affecting the cumulative trajectory. The specific formula is as follows:

(1) $$r_{n}=\frac{1+\sum_{i=2}^{n}C_{i}}{n}\times 100\%$$

In Equation (1), let represent whether the choice in the ith trial is correct. Here, $C_{i}=1$ indicates the choice is correct, and $C_{i}=0$ indicates the choice is incorrect. $r_{n}$ represents the cumulative correct choice rate after the nth trial.

The indicators for predicting visual fatigue in this study are shown in Table 1.

## 4. Data Processing and Modeling

### 4.1. Data Processing

#### 4.1.1. Data Segment Partition

Subjective visual fatigue scores before and after the experiment were normally distributed; therefore, a paired-samples *t*-test was performed. The results are presented in Table 2.

As shown in Table 2, the paired-samples *t*-test yielded a *p*-value < 0.05 for the pre- and post-experiment subjective visual fatigue scores, indicating a significant difference. Consequently, the pre-experiment state can be regarded as non-fatigued, whereas the post-experiment state represents a fatigued condition.

Before the experiment began, the participants were required to be in a state of sufficient sleep, a good mental state, and to ignore fatigue. Based on this, the data segments of the first 10 trials of the experiment were selected as a state without visual fatigue, termed “early trials”; and the data segments of the last 10 trials as a state with visual fatigue, termed “late trials”. The early-trial and late-trial labels were applied uniformly across all participants. We calculated the visual fatigue indicators for the “early trials” and “late trials” based on the content in Section 3.2.

**Figure 4:**
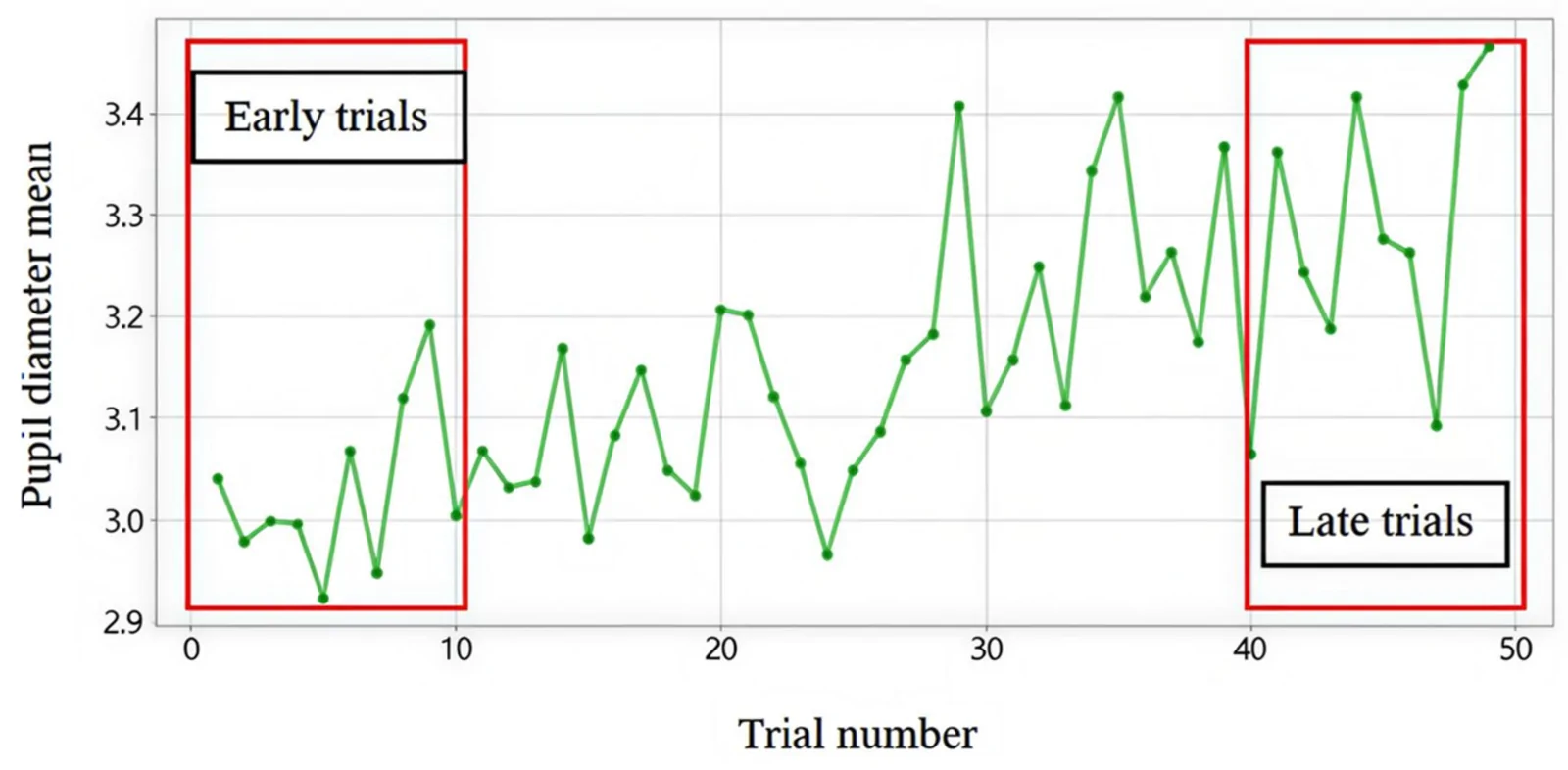
Illustration of the early-trial and late-trial partitioning strategy.

To illustrate how the early and late trial windows capture changes in visual fatigue, Figure 4 plots the mean pupil diameter across trials for a representative participant. The first 10 trials, labeled ‘early trials’, reflect the relatively non-fatigued state, whereas the last 10 trials, labeled ‘late trials’, reflect the fatigued state at the end of the session.

#### 4.1.2. Selection of Indicators

To identify features that differed between the early and late stages of the experiment, a participant-level Wilcoxon signed-rank test for each feature was conducted. Specifically, for each feature and each participant, we computed the mean value across all early trials and the mean value across all late trials, resulting in one early score and one late score per participant. The Wilcoxon signed-rank test was then applied to the 48 paired early–late observations.

Because 19 features were tested simultaneously, we controlled the false discovery rate (FDR) using the Benjamini–Hochberg procedure with a significance threshold of α = 0.05. A feature was considered statistically significant if its FDR-adjusted *p*-value was less than 0.05.

This procedure identified 10 FDR-significant features associated with visual fatigue; the results are presented in Table 3.

Although 10 features were statistically significant, several of them were highly intercorrelated and carried redundant information. Including strongly correlated predictors in the same classifier can inflate variance and reduce model interpretability. Therefore, we performed a redundancy-removal step before model training.

**Figure 5:**
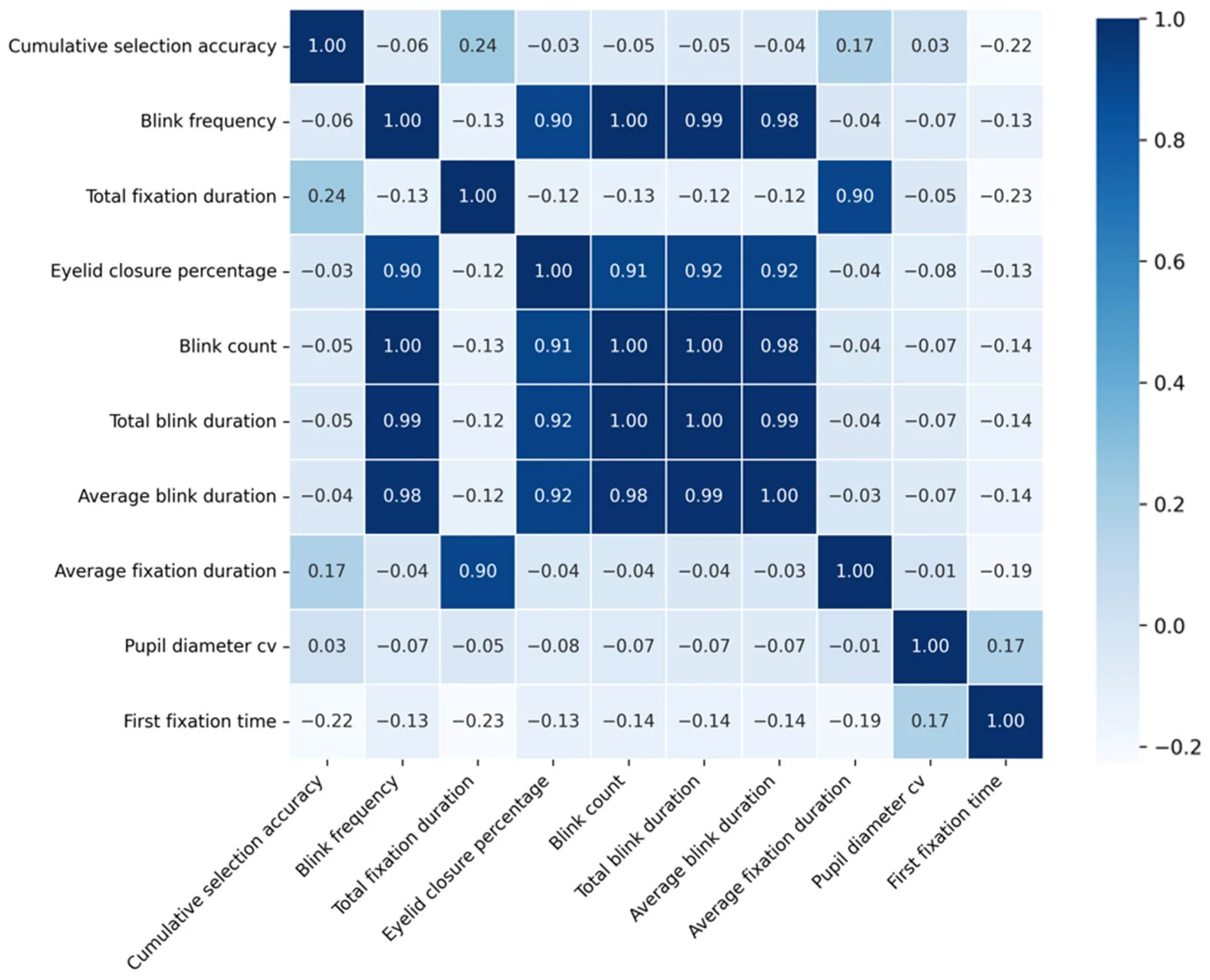
Result of Spearman correlation analysis.

We computed the Spearman correlation matrix across the combined early and late trial-level data for the ten significant features, the visualization of the result of Spearman correlation analysis is shown in Figure 5. We then scanned the features in the order of their FDR-adjusted *p*-values (from most to least significant). For any pair of features with an absolute Spearman correlation coefficient |ρ| > 0.8, the later feature in this ordering was automatically flagged for removal.

This automatic procedure produced five non-redundant features. However, we applied an adjustment based on physiological interpretability. We retained blink frequency and eyelid closure percentage—even though their pairwise Spearman correlation was ρ = 0.90. These two features capture conceptually distinct aspects of ocular fatigue: blink frequency reflects the rate of blink events, whereas eyelid closure percentage reflects the proportion of time the eyelid spends in a closed state. Both metrics have been linked to fatigue in separate streams of the oculomotor literature, and their joint inclusion allows the classifiers to exploit complementary information rather than redundant information alone.

Similarly, for the fixation duration cluster, we retained average fixation duration and removed total fixation duration, because average duration is less dependent on the number of fixations and trial length, making it a more stable and interpretable indicator of sustained attention during fatigue.

Finally, the indicators that can characterize the state of visual fatigue are selected as follows: cumulative selection accuracy, blink frequency, eyelid closure percentage, average fixation duration, pupil diameter CV, and first fixation time, a total of 6 indicators.

### 4.2. Model Building

#### 4.2.1. GBDT-LR

**Figure 6:**
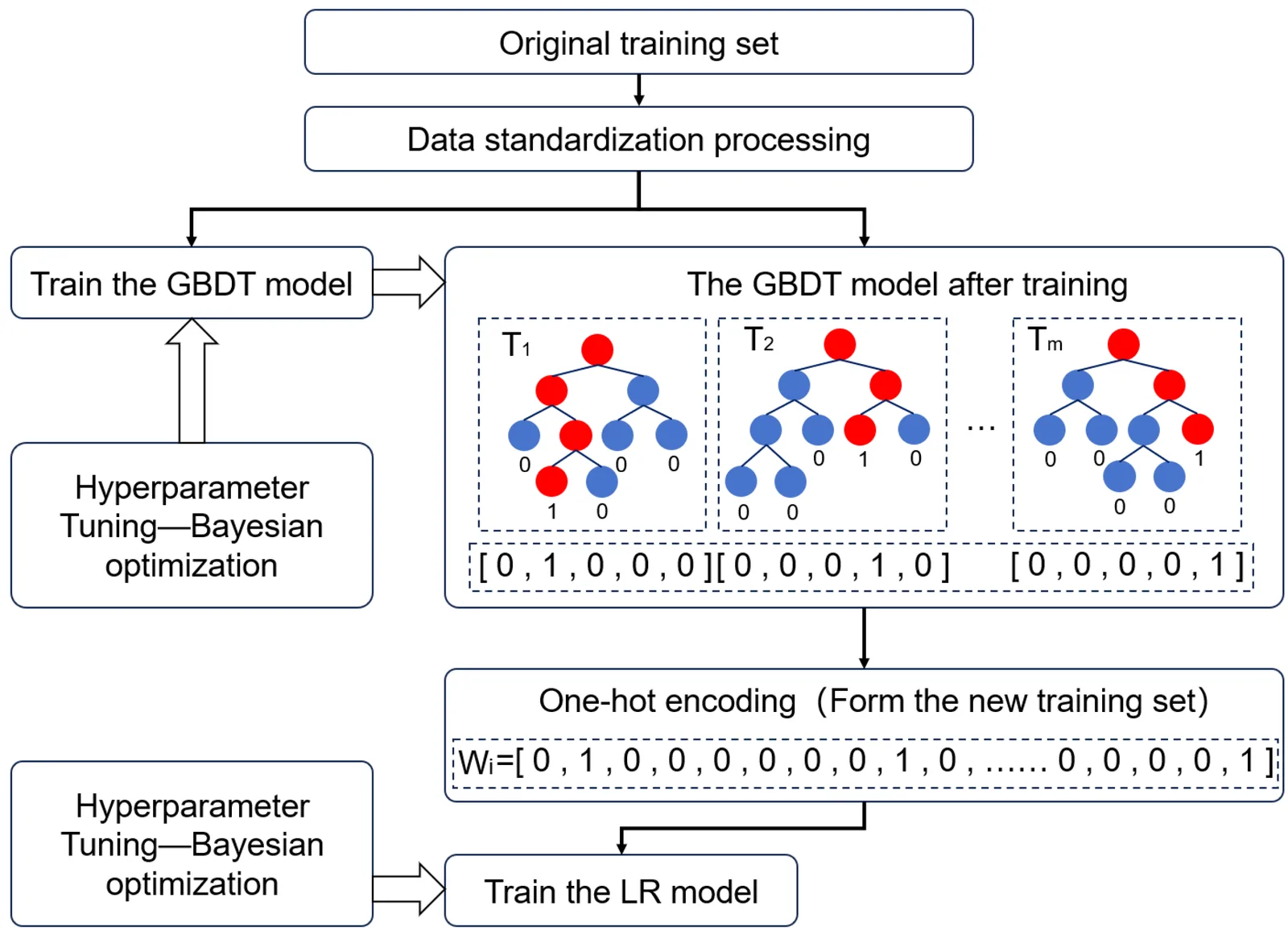
Model construction process.

LR model has low computational complexity and performs well in parallel processing data, but its linear model structure limits its model learning capability to a low level. Before applying it, it is usually necessary to carry out a certain scale of feature engineering. Considering that the GBDT model is particularly adept at detecting intricate relationships among features [42], this study proposes the GBDT-LR algorithm. The algorithm uses GBDT as the learning layer model of LR to solve the problem of low learning ability of LR and the need for large-scale feature analysis engineering [43]. In addition, the GBDT-LR algorithm is less prone to severe overfitting than deep-learning models because of its limited tree depth and sparse final logistic regression layer. This approach mitigates the risk of overfitting, reduces information loss, and augments the stability and precision of the model. Next, we will describe in detail the construction process of the GBDT-LR algorithm, as illustrated in Figure 6.

The GBDT-LR model was evaluated under the same 5-fold stratified cross-validation framework used for all other classifiers. Stratification ensured that each fold preserved the balanced proportion of early and late trials. In each fold, the data were split into a training set and a validation set, and the following steps were performed.

Firstly, the training fold was standardized. Because different eye movement features and task performance measures vary substantially in value range, features with larger scales could dominate the tree-splitting process and reduce model stability. To mitigate this, z-score standardization was applied to the training fold, and the fitted scaler was then used to transform both the training and validation folds. The standardization formula is as follows:

(2) $$z=\frac{\left(x-\mu \right)}{\sigma }$$

In Equation (2), x represents sample data, μ represents the mean, and σ represents the standard deviation.

Next, the GBDT model was trained on the standardized training fold. Bayesian optimization was applied to tune the GBDT hyperparameters, including the number of estimators, learning rate, maximum tree depth, minimum samples split, and minimum samples leaf, producing a strong classifier.

Utilizing the well-trained GBDT model, assessments were made on the standardized training fold. Instead of outputting classification probabilities, the position of the leaf nodes to which each tree’s assessment values belong is extracted as new features, forming a new dataset. During the iterative process of the GBDT algorithm, each round of iteration generated a new decision tree, and the assessment results of this tree were accumulated onto the assessments of all previous trees. This process ensures that the generation of new trees is aimed at correcting the assessment errors of the previous model. When constructing decision trees, the algorithm prioritizes selecting features that can significantly differentiate most samples for node splitting and then seeks features that can distinguish the remaining minority of samples. Consequently, the new set of features generated in this way can capture information that is discriminative for both the majority and minority of samples. This strategy of first capturing major features and then refining them to minor features is highly effective for feature engineering.

The new data was subjected to one-hot encoding to convert it into a form that can be input into the LR algorithm. This involved marking the leaf node positions to which each original training sample falls after being predicted by the GBDT model as 1 (red nodes in Figure 6), and all other leaf node positions as 0 (blue nodes in Figure 6), resulting in a sparse vector Wi that corresponded to the marked leaf node positions for each decision tree’s output. As shown in Figure 6, T1, T2, …, Tm are the m decision trees generated through m iterations during the training process of the GBDT algorithm. Assuming that T1, T2, …, Tm each have 5 leaf nodes, for the *i*-th original data sample, the assessment result of tree T1 is represented by one-hot encoding as [0, 1, 0, 0, 0], the assessment result of tree T2 is represented as [0, 0, 0, 1, 0], and so on, until the assessment result of tree Tm is represented as [0, 0, 0, 0, 1]. Therefore, the sparse vector Wi for this original data sample is formed as [0, 1, 0, 0, 0, 0, 0, 0, 1, 0, …, 0, 0, 0, 0, 1], which is the process of forming the sparse vector Wi. All sparse vectors, along with the corresponding classifications of the original training samples, constitute the new training set for the LR algorithm.

The new training set is then used to train the LR model. The LR hyperparameter C was also selected via Bayesian optimization within the same inner cross-validation loop. After training, the standardized validation fold was passed through the same fitted GBDT to extract leaf node indices, one-hot encoded using the encoder fitted on the training fold, and finally evaluated by the trained LR model. This fold-level training and validation procedure was repeated for all five folds, and the reported GBDT-LR performance was the mean ± standard deviation across the five validation folds.

#### 4.2.2. Performance of the Model

In this study, 6 supervised classifiers were trained using the 6 selected features as inputs: LR, SVM, RF, RF-SVM, GBDT, and GBDT-LR. All models were evaluated under a unified 5-fold stratified cross-validation (CV) framework. The evaluation metrics include accuracy, precision, recall, F1-score, and AUC.

**Figure 7:**
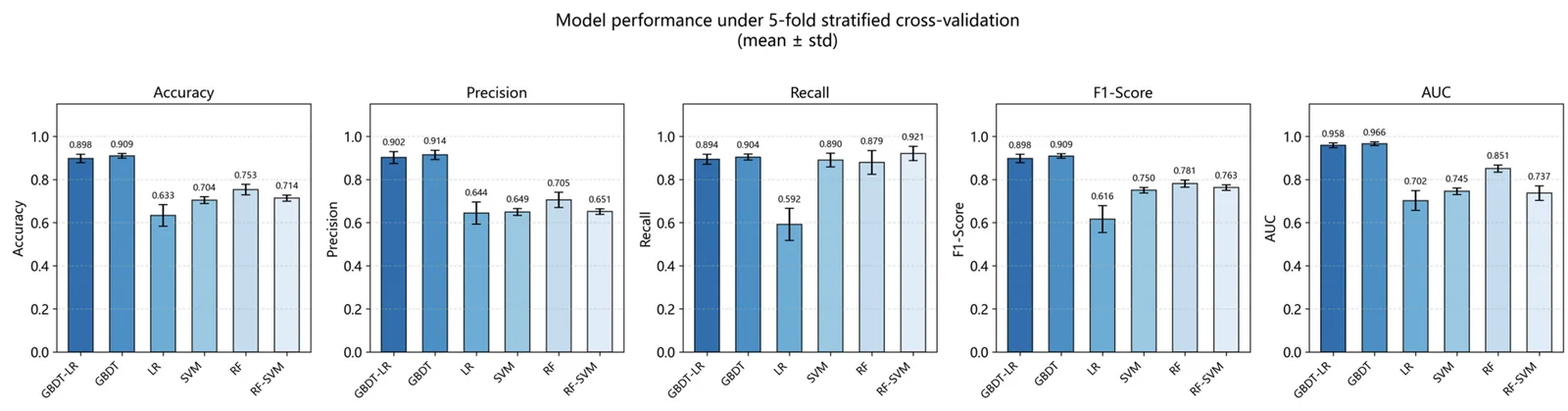
The performance comparison of various models.

The complete results are summarized in Table 4 and Figure 7. Overall, tree-based ensemble methods achieved the highest classification performance. GBDT yielded the best mean accuracy (0.9094 ± 0.0108) and the highest AUC (0.9660 ± 0.0086), followed closely by GBDT-LR (accuracy = 0.8979 ± 0.0197, AUC = 0.9582 ± 0.0112). Linear logistic regression performed worst (accuracy = 0.6333 ± 0.0499), suggesting that the relationship between the selected features and fatigue state is non-linear. SVM, RF, and RF-SVM fell in the intermediate range.

Because GBDT and GBDT-LR were the top two performing models, we conducted a paired statistical comparison between them. We applied both the Wilcoxon signed-rank test and the paired *t*-test to the 5-fold level scores for each metric. Neither test reached statistical significance at α = 0.05 for any metric (all Wilcoxon *p*-values ≥ 0.125, all paired *t*-test *p*-values ≥ 0.140). Thus, although GBDT had the highest mean accuracy, its advantage over GBDT-LR was not statistically significant under this cross-validation scheme.

Meanwhile, both models consistently outperformed LR, SVM, RF, and RF-SVM by a substantial margin. Given that GBDT-LR inherits the low computational complexity and strong linear optimization capability of LR while automatically generating high-order feature interactions through GBDT, this study still recommends GBDT-LR as an effective hybrid evaluation framework for visual fatigue assessment.

## 5. Discussion

**General discussion.** The results indicate that eye movement features and task performance can reflect the occurrence of visual fatigue. Previous studies have shown that eye movement behavior can reflect valuable information about an individual’s cognitive processes, visual attention, and emotional arousal [44]. Therefore, eye movement features are commonly used indicators for assessing visual fatigue. In this study, the final retained indicators—cumulative selection accuracy, blink frequency, eyelid closure percentage, average fixation duration, pupil diameter cv, and first fixation time—collectively capture oculomotor slowing, autonomic pupillary instability, eyelid closure behavior, and declining task performance. In addition, mental fatigue is often manifested by a reduction in motivation for effortful activities and impaired task performance [45,46]. Thus, using the task performance indicator “Cumulative selection accuracy” to evaluate visual fatigue is reasonable. A visual fatigue assessment method that combines eye movement features and task performance contains richer information [30] and can more comprehensively reflect the occurrence of visual fatigue.

The results also indicate that the assessment model constructed in this study can accurately predict the occurrence of visual fatigue during eye-controlled interaction based on eye movement characteristics and task performance indicators, combined with the GBDT-LR algorithm and Bayesian optimization for hyperparameter tuning. This is because the proposed method combines the advantages of the GBDT algorithm and the LR algorithm, which can effectively handle high-dimensional and complex data [17]. The relationship between eye movement data, task performance data, and visual fatigue is often complex and not simply linear, which explains the lower accuracy of LR. Under the unified 5-fold stratified cross-validation framework, both GBDT and GBDT-LR achieved the highest level of performance (GBDT: AUC = 0.9660 ± 0.0086; GBDT-LR: AUC = 0.9582 ± 0.0112). No significant difference was observed between these two models across any metric, suggesting that their discriminative capacities are comparable. Meanwhile, both models consistently outperformed LR, SVM, RF, and RF-SVM by a substantial margin, particularly in terms of AUC and F1-score. The lower accuracy of RF and RF-SVM may be due to the random feature selection of RF, which can neglect important fatigue-related features. The higher recall of RF-SVM is believed to reflect the fact that some fatigue-representative features are more pronounced in positive samples, allowing the SVM component to perform better on those cases. The strong performance of GBDT-LR arises because GBDT captures the main features that significantly distinguish visual fatigue in most samples through stepwise optimization and then refines the secondary features that distinguish fatigue in a smaller subset of samples through subsequent trees. Combining this with the low computational complexity and strong linear optimization ability of LR, GBDT-LR achieves accurate and efficient visual fatigue assessment. In addition, through Bayesian optimization, hyperparameter tuning is performed more efficiently, improving the accuracy and stability of the assessment [47].

**Theoretical interpretation of the selected indicators.** The eye movement and performance features retained in the final model can be linked to the physiological mechanisms reviewed in Section 2.2. Increased first fixation time is consistent with slowed initial orienting and greater accommodative/vergence effort during fatigue. Decreased average fixation duration may reflect a more fragmented scanning pattern: as fatigue accumulates, participants have difficulty maintaining sustained gaze and produce shorter, more frequent fixations. Increased blink frequency and increased eyelid closure percentage reflect reduced spontaneous blinking and increased ocular surface exposure, which are characteristic of dry-eye-related ocular fatigue. Decreased pupil diameter CV is interpreted as reduced autonomic variability under sustained visual-cognitive load, suggesting a less flexible pupillary control system, although ambient light and cognitive arousal also influence pupil dynamics. Finally, declining cumulative selection accuracy captures the functional consequence of visual fatigue on task performance. By combining these complementary indicators, the model approximates a multidimensional construct of visual fatigue rather than a single physiological surrogate.

**Distinguishing visual fatigue from related constructs.** It is important to note that pupil dilation and some task performance changes can also be elicited by mental workload or cognitive stress, which share autonomic and performance signatures with visual fatigue. In the present study, the induced-fatigue paradigm, the use of ocular-specific symptom items, and the convergence of multiple eye movement indicators help the model distinguish visual fatigue rather than general cognitive load. Nevertheless, perfect construct separation is difficult without concurrent workload or stress measures, and future work should include validated scales (e.g., NASA-TLX workload, State-Trait Anxiety Inventory) to disentangle these constructs.

**Real-time deployment considerations.** Although Bayesian optimization is computationally expensive, it is performed offline during model training and therefore does not contribute to online inference latency. The online pipeline—feature extraction from the eye tracker followed by a forward pass through the trained GBDT-LR model—is lightweight. To ensure real-time operation in consumer eye-controlled interaction systems, several engineering strategies can be adopted: limiting tree depth and ensemble size, reducing the feature set to the most discriminative indicators, quantizing model parameters for edge deployment, warm-starting Bayesian optimization when retraining, selecting a latency–accuracy operating point that meets the application budget, and deploying via optimized inference engines. These strategies make the framework feasible for real-time fatigue monitoring without requiring high-end hardware.

## 6. Conclusions

This study proposed a visual fatigue assessment method based on Bayesian-optimized GBDT-LR for eye-controlled interaction, comprehensively taking into account readily monitorable information such as eye movement characteristics and task performance. The results indicated that eye movement characteristics and task performance can effectively characterize the state of visual fatigue. Moreover, the proposed assessment model was compared with five other methods (GBDT, LR, SVM, RF, RF-SVM) under a unified 5-fold stratified cross-validation framework. Both GBDT and GBDT-LR reached the highest level of fatigue-detection performance, and the two models performed comparably. Both models consistently outperformed LR, SVM, RF, and RF-SVM by a substantial margin. Considering that GBDT-LR inherits the low computational complexity and strong linear optimization capability of LR while automatically generating high-order feature interactions through GBDT, this study still recommends GBDT-LR as an effective hybrid evaluation framework for visual fatigue assessment in eye-controlled interaction. This study provided an effective method for the assessment of visual fatigue and laid a research foundation for optimizing the user experience of eye-controlled interaction, promoting the sustainable development of eye-controlled technology.

**Limitations.** Several limitations should be acknowledged. First, model validation used 5-fold stratified cross-validation with stratification by fatigue state (early/late) but not by participant. Although the scaler and Bayesian hyperparameter optimizer were fitted exclusively within each training fold to avoid preprocessing leakage, trials from the same participant could still appear in both the training and validation folds. Consequently, the reported performance may partly reflect participant-specific oculomotor patterns and should be interpreted as an internal benchmark rather than as a fully realistic estimate of cross-participant generalizability. Second, the binary early/late-trial labeling strategy simplifies the inherently continuous and non-linear process of fatigue accumulation and ignores individual differences in fatigue susceptibility and adaptation. Third, the sample is homogeneous and modest in size (48 university students, predominantly young and male), limiting generalizability to older adults, other genders, or clinical populations. Fourth, environmental conditions such as ambient illuminance were not continuously monitored, which may confound fatigue-related changes in pupil diameter. Fifth, subjective fatigue ratings were collected only before and after the experiment, providing a coarse global anchor rather than continuous tracking of fatigue dynamics. Finally, although participants completed a standardized practice session and the late-trial performance decline is consistent with fatigue accumulation, we cannot formally rule out the contribution of learning effects without additional statistical tests or a control condition.

**Future work.** Future work will address these limitations in several directions. To obtain more realistic performance estimates and assess cross-participant generalizability, we will adopt stricter validation protocols, such as Leave-One-Subject-Out (LOSO) cross-validation, subject-stratified k-fold cross-validation, and external validation on independent samples. To account for individual differences, we will collect larger, demographically diverse samples and explore personalized models that adapt to age, gender, and baseline oculomotor characteristics. To track fatigue dynamics without disrupting the task, we will incorporate non-intrusive subjective ratings, such as intermittent visual-analog scales or experience-sampling probes, into the experimental procedure. Finally, we will move beyond binary fatigue detection toward multi-class fatigue-level assessment (e.g., none, mild, moderate, severe) and model the cumulative temporal evolution of visual fatigue using time-series approaches, thereby supporting adaptive interventions that respond to emerging fatigue before performance degrades.

## Figures and Tables

**Table 1:** Visual fatigue indicators.

Category	Indicator	Description
Subjective questionnaire	Subjective score	Used to evaluate the degree of subjective visual fatigue.
Eye movement data	Fixation count	The number of times the eye stays in the AOI (Area of Interest) area and makes a fixation in a trial.
	Total fixation duration (ms)	The sum of the duration of all fixations in a trial.
	Average fixation duration (ms)	The average of all fixation durations in a trial.
	First fixation time (ms)	The time taken from the time the stimulus was presented to the time the sight first entered the AOI area in a trial.
	Saccade count	The number of times the eye moves from one fixation point to another in a trial.
	Total saccade duration (ms)	The sum of the duration of all saccades in a trial.
	Average saccade duration (ms)	The average duration of all saccades in a trial.
	Average saccade amplitude (°)	The average of the angles at which the eye moves from one fixation point to another in a trial.
	Average saccade velocity (pixels·ms^−1^)	The average of the speed at which the eye moves from one fixation point to another in a trial.
	Blink count	The total number of blinks that occur in a trial.
	Blink frequency	The number of blinks per unit time in a trial.
	Total blink duration (ms)	The sum of all blink durations in a trial.
	Average blink duration (ms)	The average of all blink durations in a trial.
	Eyelid closure percentage (%)	The amount of time it takes for the eye to close a certain percentage (according to P80) in a trial.
	Pupil diameter mean (mm)	The average size of the pupil diameters of the left and right eyes in a trial.
	Pupil diameter std (mm)	The square root of the mean square of the deviation between the pupil diameter of each data point and the mean pupil diameter in a trial.
	Pupil diameter cv	Ratio of standard deviation of pupil diameter to mean pupil diameter in a trial.
Task performance data	Selection reaction time (ms)	The time taken from the presentation of the stimulus to the first entry of the gaze into the target letter range in a trial.
	Cumulative selection accuracy (%)	The percentage of correctly selected trials up to the current number of trials. The cumulative selection accuracy for the first trial is set to be 100%.

**Table 2:** Paired-samples *t*-test results for subjective visual fatigue scores.

Paired-Samples *t*-Test	Mean ± SD		Difference	t	*p*
	Pre-Experiment	Post-Experiment			
Pre-experiment vs. Post-experiment	7.458 ± 5.570	13.354 ± 5.334	−7.833	−6.205	<0.001

**Table 3:** Participant-level Wilcoxon results with FDR correction.

Indicator	Descriptive Analysis (Mean)		Wilcoxon Signed-Rank Test			
	Early Trials	Late Trials	W	*p*	FDR-p	Direction
Fixation count	10.952	9.956	412	0.071	0.123	Decrease
Total fixation duration	1679.236	1452.986	210	<0.001	<0.001	Decrease
Average fixation duration	157.875	140.835	306	0.003	0.008	Decrease
First fixation time	1157.689	1292.168	336	0.009	0.017	Increase
Saccade count	5.954	6.223	496	0.345	0.410	Increase
Total saccade duration	245.104	263.038	504	0.389	0.435	Increase
Average saccade duration	40.434	40.158	540	0.629	0.629	Decrease
Average saccade amplitude	1.881	1.854	455	0.176	0.223	Decrease
Average saccade velocity	225.124	221.550	448	0.154	0.209	Decrease
Blink count	0.227	0.408	216.5	<0.001	<0.001	Increase
Blink frequency	4.354	8.554	163.5	<0.001	<0.001	Increase
Average blink duration	18.095	33.136	265	0.001	0.002	Increase
Total blink duration	26.545	48.368	232	<0.001	<0.001	Increase
Eyelid closure percentage	0.536	1.267	207.5	<0.001	<0.001	Increase
Pupil diameter mean	3.607	3.637	417	0.122	0.186	Increase
Pupil diameter std	0.123	0.118	419	0.127	0.186	Decrease
Pupil diameter cv	0.034	0.031	336	0.009	0.017	Decrease
Selection reaction time	288.860	293.804	538	0.615	0.629	Increase
Cumulative selection accuracy	84.046	74.486	79	<0.001	<0.001	Decrease

**Table 4:** Model performance comparison (mean ± std) for 6 classifiers.

	Accuracy	Precision	Recall	F1-Score	AUC
GBDT-LR	0.8979 ± 0.0197	0.9019 ± 0.0273	0.8938 ± 0.0238	0.8975 ± 0.0192	0.9582 ± 0.0112
GBDT	0.9094 ± 0.0108	0.9142 ± 0.0217	0.9042 ± 0.0136	0.9090 ± 0.0101	0.9660 ± 0.0086
LR	0.6333 ± 0.0499	0.6442 ± 0.0517	0.5917 ± 0.0742	0.6161 ± 0.0626	0.7023 ± 0.0460
SVM	0.7042 ± 0.0158	0.6494 ± 0.0158	0.8896 ± 0.0318	0.7504 ± 0.0125	0.7453 ± 0.0149
RF	0.7531 ± 0.0240	0.7050 ± 0.0354	0.8792 ± 0.0554	0.7808 ± 0.0168	0.8507 ± 0.0165
RF-SVM	0.7135 ± 0.0143	0.6513 ± 0.0131	0.9208 ± 0.0334	0.7626 ± 0.0128	0.7372 ± 0.0336

## Data Availability

Data is contained within the article.

## Abbreviations

The following abbreviations are used in this manuscript:

GBDT-LR	Gradient Boosting Decision Tree–Logistic Regression
GBDT	Gradient Boosting Decision Tree
LR	Logistic Regression
SVM	Support Vector Machine
RF	Random Forest
RF-SVM	Random Forest–Support Vector Machine
VFS	Visual Fatigue Scale
SSQ	Simulator Sickness Questionnaire
ECG	Electrocardiography
EOG	Electro-oculography
EMG	Electromyography
EEG	Electroencephalography
PVT	Psychomotor Vigilance Task
ISO	International Organization for Standardization
PERCLOS	Percentage of Eyelid Closure over the Pupil over Time
NN	Normal to Normal
LF	Low-Frequency
BF	Blink Frequency
YF	Yawn Frequency
HNF	Head Nod Frequency
LSTM	Long Short-Term Memory
RNN	Recurrent Neural Network
LCD	Liquid Crystal Display
VDT	Visual Display Terminal
SDK	Software Development Kit
I-VT	Identification by Velocity Threshold
CV	Coefficient of Variation
AOI	Area of Interest
P80	80% Eyelid Closure Threshold
SD	Standard Deviation
AUC	Area Under the Curve
LOSO	Leave-One-Subject-Out
NASA-TLX	NASA Task Load Index
